# Wnt-5a induces the conversion of silent to functional synapses in the hippocampus

**DOI:** 10.3389/fnmol.2022.1024034

**Published:** 2022-10-28

**Authors:** Carla Álvarez-Ferradas, Mario Wellmann, Koyam Morales, Marco Fuenzalida, Waldo Cerpa, Nibaldo C. Inestrosa, Christian Bonansco

**Affiliations:** ^1^Facultad de Ciencias, Centro de Neurobiología y Fisiopatología Integrativa (CENFI), Instituto de Fisiología, Universidad de Valparaíso, Valparaíso, Chile; ^2^Escuela de Fonoaudiología, Facultad de Medicina, Universidad de Valparaíso, Valparaíso, Chile; ^3^Centro de Excelencia en Biomedicina de Magallanes (CEBIMA), Universidad de Magallanes, Punta Arenas, Chile; ^4^Departamento de Biología Celular y Molecular, Facultad de Ciencias Biológicas, Center of Aging and Regeneration UC (CARE-UC), Pontificia Universidad Católica de Chile, Santiago, Chile

**Keywords:** Wnt signaling, silent synapses, hippocampus, development, NMDA postsynaptic currents, AMPA receptors

## Abstract

Synapse unsilencing is an essential mechanism for experience-dependent plasticity. Here, we showed that the application of the ligand Wnt-5a converts glutamatergic silent synapses into functional ones by increasing both α-amino-3-hydroxy-5-methyl-4-isoxazole propionic acid (AMPA) and N-methyl-D-aspartate (NMDA) currents (I_AMPA_ and I_NMDA_, respectively). These effects were mimicked by the hexapeptide Foxy-5 and inhibited by secreted frizzled-related protein sFRP-2. I_NMDA_ potentiation was produced by increased synaptic potency, followed by an increase in the probability of release (Pr), even in the presence of 7-nitro-2,3-dioxo-1,4-dihydroquinoxaline-6-carbonitrile (CNQX). At a longer time of Wnt-5a exposure, the Pr increments were higher in I_NMDA_ than in I_AMPA_. In the presence of NMDAR inhibitors, Wnt-5a-induced conversion was fully inhibited in 69.0% of silent synapses, whereas in the remaining synapses were converted into functional one. Our study findings showed that the Wnt-5a-activated pathway triggers AMPAR insertion into mammalian glutamatergic synapses, unsilencing non-functional synapses and promoting the formation of nascent synapses during the early postnatal development of the brain circuits.

## Introduction

Most glutamatergic synapses are ineffective or silent at resting potential during the early stages of brain development. Their conversion into functional synapses is critical for the normal maturation of the central nervous system (CNS) (Voronin and Cherubini, 2004; Kerchner and Nicoll, 2008). In the postsynaptic membrane, silent synapses express N-methyl-D-aspartate receptors (NMDARs) but not α-amino-3- hydroxy-5-methyl-4-isoxazole propionic acid receptors (AMPARs), which makes them unresponsive to the binding of glutamate at resting membrane potential due to the voltage-dependent blockade of the NMDA-mediated current by Mg^2+^ (Liao et al., 1995, 2001; Busetto et al., 2008). Postsynaptic AMPAR insertion is the primary mechanism mediating silent synapses’ conversion into functional synapses (Malenka, 2003; Groc et al., 2006). NMDARs in silent synapses of the immature hippocampus are formed mainly by heterotetramers rich in ifenprodil-sensitive GluN2B subunits (Sheng, 2001; Barria and Malinow, 2002; Yasuda and Mukai, 2015). Although several cell signaling molecules can induce synaptic potentiation in mature synapses through glutamate receptor trafficking and insertion (Escobar et al., 2003; Christopherson et al., 2005; Allen et al., 2012), only some of them have been implicated in the conversion of silent synapses in early development (Li et al., 2010; Cabezas and Buno, 2011; Wang et al., 2013). Secreted Wnt glycoproteins play critical morphogenetic roles, including neuronal migration, axon pathfinding, dendritogenesis, and synaptogenesis, and are widely expressed during brain development (Farias et al., 2009, 2010; Cerpa et al., 2010; Chen et al., 2013; Rosso and Inestrosa, 2013; Inestrosa and Varela-Nallar, 2015). Wnt ligands bind to Frizzled (Fz) and tyrosine kinase-like orphan receptor RoR2, which can trigger different signaling cascades, including a non-canonical Wnt/Ca^2+^ pathway that increases intracellular Ca^2+^ levels and activates calcium-sensitive kinases CaMKII and PKC (Cerpa et al., 2011, 2015; Munoz et al., 2014). Previously, we showed that this non-canonical Wnt-5a pathway exerts a postsynaptic effect that increases the amplitude of excitatory postsynaptic currents (EPSCs) mediated by both AMPA and NMDA currents (I_AMPA_ and I_NMDA_, respectively) in the CA3-CA1 synapses of young rat hippocampal slices (Cerpa et al., 2010, 2011; Farias et al., 2010). This change was more pronounced for I_NMDA_ than I_AMPA_, which was associated with an increase in intracellular Ca^2+^ and SNARE-dependent trafficking, which increased the number and clustering of GluN2B-rich receptors (Dinamarca et al., 2008; Farias et al., 2009). However, although Wnt signaling has been positioned as a critical pathway in the formation and maintenance of CNS synapses, whether the ligand Wnt-5a contributes to the conversion of silent synapses to functional synapses is still an unanswered question.

By recording at a single glutamatergic synapse in the hippocampal formation of neonatal rats, we showed that Wnt-5a converts silent glutamatergic synapses into functional synapses by increasing I_AMPA_, which is followed by an increase in I_NMDA_. These plastic changes are triggered by Ca^2+^-dependent mechanisms, whose influx requires GluN2B-containing NMDARs. Altogether, we showed that the ligand Wnt-5a induces the conversion of silent synapses during neonatal development, promoting a concerted mechanism that balances receptors insertion at the postsynaptic neuron and increases in synaptic connectivity.

## Experimental procedures

### Ethics statement

All procedures relating to animal experimentation were in strict accordance with National Institutes of Health (USA) guidelines for the use of experimental animals, which were approved by the Ethics and Biosafety Committee of the Universidad de Valparaíso and the Institutional Animal Experimentation Ethics Board and the Science Council (FONDECYT) of Chile. Furthermore, the greatest of efforts were made to minimize the number of animals used and their suffering.

### Slices and electrophysiology

Acute slices from the dorsal hippocampus were obtained from 5- to 10-day-old male and female Sprague-Dawley rats. Each rat was anesthetized with isoflurane and decapitated, and the brain was rapidly removed through craniotomy and submerged in cold (less than 4°C) artificial cerebrospinal fluid (ACSF, in mM: 124.0 NaCl, 2.7 KCl, 1.25 KH_2_PO_4_, 2.0 MgSO4, 26.0 NaHCO_3_, 2.0 CaCl_2_, 10.0 glucose) gassed with a mixture of 95% O_2_ and 5% CO_2_ (pH 7.4). Transverse hippocampal slices (350 μm) were cut with a vibroslice microtome (VSL, WPI Inst., USA) and incubated in ACSF (> 1 h). All experiments were performed at room temperature (22–24°C) in a recording chamber (2 ml) and fixed to an upright microscope stage (FN100 IR; Nikon Inc., Japan) equipped with infrared and differential interference contrast imaging devices (40X). Slices were perfused with ACSF in the presence of the GABAergic antagonist picrotoxin (PTX; 10 μM). Patch-clamp recordings from CA1 pyramidal neurons in a whole-cell voltage-clamp configuration was obtained as previously described (Cerpa et al., 2010; Bonansco et al., 2011). The intracellular recording solution contained (in mM) 100.0 cesium gluconate, 10.0 tetraethylammonium, 10.0 4-(2-hydroxyethyl)-1-piperazine-ethane sulfonic acid, 1.0 MgCl_2_, 10.0 ethylene-bis-(oxonitrilo) tetraacetate and 4.0 sodium salt (Na-ATP) at pH 7.2 (adjusted with CsOH). Minimal bipolar stimulation of Schaffer collaterals (200 μs duration, 2.0 s^–1^) was performed with a septum theta patch-like pipette, gently placed in the *stratum radiatum* around the dendritic tree of the recorded cell (<100 μm). The activation of a putative single synapse was assumed when all-or-none failures or minimally evoked excitatory postsynaptic current (meEPSC) successes were achieved and corroborated by applying a single-synapse test (see Supplementary Figure 1; Hsia et al., 1998). Events that exhibited an amplitude of more than three standard deviations (SDs) above the baseline current were considered successes, while any event with an amplitude of less was considered a failure. To minimize the contribution of postsynaptically mediated plasticity (Malinow and Tsien, 1990), a series of 50–150 stimuli were delivered discontinuously (1.0–0.3 Hz) by minimal stimulation of Shaffer collaterals, setting the baseline recordings of meEPSCs during 10 min after the achieved of the whole-cell configuration. Single synapses were considered silent when meEPSCs were evoked only at + 40 mV (I_NMDA_), even when employing paired-pulse protocols (Hanse and Gustafsson, 2001). Synaptic efficacy was estimated by averaging the peak amplitude of all responses (failures included), while synaptic potency was obtained by averaging the peak amplitude of successful responses. The I_NMDA_ was measured 40 ms after the stimulus artifact, whereas the I_AMPA_ was measured at the EPSC peak. The probability of release (Pr) was estimated by obtaining the ratio between the number of successes and the total number of stimuli.

### Drugs

Recombinant Wnt-5a ligand (rWnt-5a), Wnt-5a-derived peptide Foxy-5 and soluble Frizzled-related protein-2 (sFRP-2) were used at concentrations of 6.5 nM, 50 μM and 25 nM, respectively, and purchased from R&D Systems. The competitive NMDAR antagonist 2-amino-5-phosphonopentanoic acid (APV; 20 μM) and the competitive AMPA/kainate receptor antagonist 7-nitro-2,3-dioxo-1,4-dihydroquinoxaline-6-carbonitrile (CNQX; 20 μM) were dissolved in water. The GluN2B-subunit-containing NMDAR selective antagonist ifenprodil (10 μM) and a non-competitive NMDAR antagonist that physically blocks ion permeation, MK-801 (40 μM), were dissolved in DMSO (0.01%), whereas PTX (10 μM) was dissolved in ethanol and added to ACSF.

### Statistical analysis

Statistical analysis was performed using Student’s two-tailed *t*-test. In addition, an analysis of variance (ANOVA) with Bonferroni correction in the *post hoc* analysis was performed for multiple “group” comparisons. Significance was set at *p* ≤ 0.05 (*). Data are expressed as the mean ± the standard error of the mean (SEM).

## Results

### Wnt-5a ligand induces the conversion of silent synapses by increasing I_AMPA_ and I_NMDA_

**FIGURE 1:**
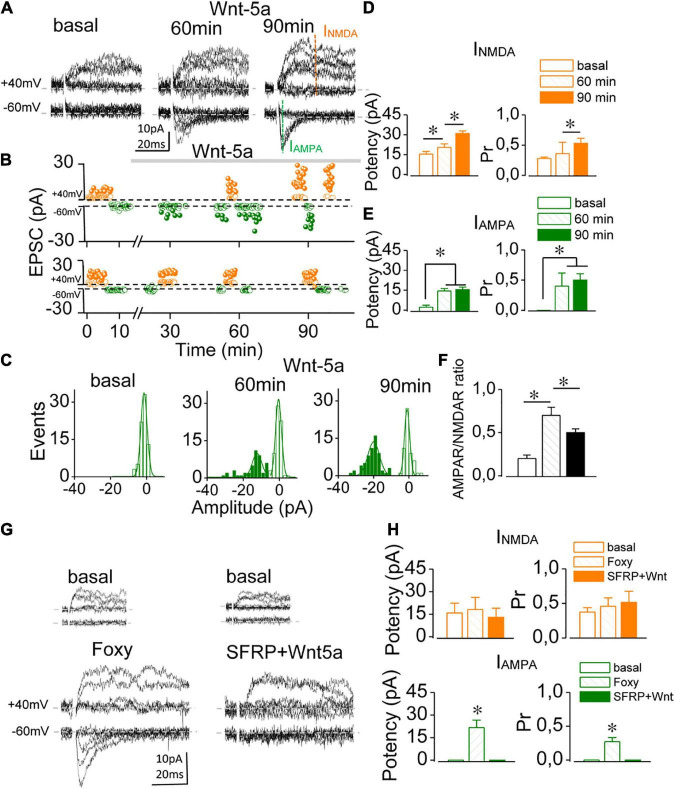
Wnt-5a induces the functional conversion of single silent synapses. **(A)** Superimposed consecutive meEPSC traces (10 sweeps) recorded at –60 and + 40 mV in a single silent synapse before (basal) and 60 and 90 min after Wnt-5a perfusion. **(B)** Time course of the changes in meEPSC amplitude corresponding to failures (empty circles) and successes (solid circles), recorded at + 40 mV (orange) and –60 mV (green) of Hp in the presence of Wnt-5a **(upper panel)** and ligand-free control **(lower panel)**. Note that from 30 min with the ligand some I_AMPA_ begin to appear. **(C)** Representative cumulative histogram of meEPSC amplitude at –60 mV before (basal) and after 60 and 90 min of Wnt-5a perfusion showing failures (white bars) and successes (green bars). **(D,E)** The average change in synaptic potency and the Pr at baseline and after 60 and 90 min of Wnt-5a at + 40 mV (orange bars) and –60 mV (green bars). **(F)** Average change in the I_AMPAR_/I_NMDAR_ ratio obtained at baseline and after Wnt-5a perfusion. **(G)** Superimposed meEPSC traces recorded at –60 and + 40 mV from two representative silent synapses before (insert) and 60 min after Foxy-5 perfusion **(left)** or sFRP-2 plus Wnt-5a **(right)**. **(H)** The average change in synaptic potency and the Pr at baseline and 60 mV after Foxy-5 or sFRP-2 plus Wnt-5a treatment was at + 40 mV (orange bars) and –60 mV (green bars). Statistics: Error bars indicate ± SEM, one-way ANOVA test (*p < 0.05).

Although several lines of evidence show that the non-canonical ligand Wnt-5a can induce potentiation in mature, functional glutamatergic synapses (Cerpa et al., 2015, 2016; McQuate et al., 2017), whether it has a similar effect in immature, silent synapses have not been elucidated. Silent synapses were recognized by the absence of AMPAR-mediated meEPSCs at an Hp of –60 mV and the presence of NMDAR-mediated EPSCs once freed from Mg^2+^ blockage at a depolarized Hp of + 40 mV (Figure 1A), while functional synapses displayed responses at both Hp (Supplementary Figure 2). Baseline meEPSCs recorded at Hp of + 40 mV exhibited (Figure 1B, failures and successes empty and solid orange circles, respectively) with a synaptic potency of 15.41 ± 1.89 pA and a Pr of 0.28 ± 0.09 (*n* = 5; Figure 1D), whereas only failures (empty green circles) were observed at an Hp of –60 mV. In presence of Wnt-5a the number and amplitude of both failures and successes recorded at –60 and + 40 mV of Hp changing as a function of time (Figure 1B, upper graph), whereas in ligand free control no change was observed (Figure 1B, lower graph). After 60 min of continued perfusion with Wnt-5a, I_AMPA_-mediated EPSCs began to occur in response to minimal stimulation at –60 mV (Figure 1B, solid green circles), increasing both the amplitude and the number of successes. The amplitude histogram of I_AMPA_-mediated EPSCs shows two peaks, one centered at 0 pA (i.e., failures) and one at 16.07 ± 8.1 pA (> 100 trials, Figure 1C) (i.e., successes). After 90 min of treatment, successes showed a peak of 20.87 ± 2.1 pA, while failures remained identical to the baseline noise (Figure 1C). After 60 min, the synaptic potency of I_AMPA_ increased respect to basal (2.78 pA ± 0.80; 60 min: 14.37 ± 1.64 pA; *p* = 0.0002) and after 90 min were 15.04 ± 1.74; *n* = 5; *p* = 0.0001, as did the Pr (from zero up to 0.41 ± 0.21 at 60 min; *p* = 0.0005 and 0.52 ± 0.105 at 90 min; *n* = 5; *p* = 0.0001). In addition, the synaptic potency of I_NMDA_ was higher at 90 min than at 60 min (20.38 ± 2.84 pA vs. 30.85 ± 2.03 pA; *p* = 0.017; *n* = 5, Figures 1D,E). Furthermore, the mean Pr values increased with exposure to Wnt-5a (60 min: 0.36 ± 0.03 vs. 90 min: 0.53 ± 0.05; *n* = 5; *p* = 0.033; Figure 1D). The AMPAR/NMDAR ratio increased from 0.20 ± 0.04 to 0.74 ± 0.09 (*n* = 5; *p* = 0.001) at 60 min of perfusion and then decreased to 0.48 ± 0.03 at 90 min (Figure 1F; *p* = 0.005), suggesting that I_NMDA_ continues to rise after AMPA-mediated conversion has occurred.

Next, to establish the specificity of the Wnt-5a effect on silent synapses, we used a specific mimicking peptide, Foxy-5 (Varela-Nallar et al., 2010), and sFRP-2, which binds to Wnt, preventing its action on Frizzled receptors. In silent synapses, treatment with Foxy-5 incorporated I_AMPA_ measured at 60 min, reaching 21.71 ± 4.87 pA with a Pr of 0.27 ± 0.06 (*n* = 5; *p* = 0.00027; Figures 1G,H), while the NMDAR currents remained unchanged (basal: 15.88 ± 6.52 pA vs. Foxy-5: 18.12 ± 8.03 pA; *n* = 5; *p* = 0.081). Similar effects were observed in functional synapses (see Supplementary Figure 2). Before assessing specific effects on Wnt-5a, we discard any effect of SFRP-2 on silent synapses, which remained unchanged during continuous perfusion with the Wnt scavenger (see Supplementary Figure 6). After 60 min of Wnt-5a plus sFRP-2 perfusion, the conversion was completely inhibited (Figure 1G). The synaptic potency of NMDAR-mediated EPSCs remained unchanged (basal 13.07 ± 5.12 pA vs. sFRP plus Wnt-5a: 12.95 ± 6.10 pA; *n* = 3; *p* = 0.065; Figure 1H). Additionally, under Wnt 5A plus sFRP-2, the Pr remained unchanged (basal: 0.37 ± 0.05 vs. sFRP-2 plus Wnt-5a: 0.61 ± 0.10; *n* = 3; *p* = 0.073). These results suggest that Wnt-5a converts silent synapses into functional ones in the immature hippocampus, increasing the I_AMPA_ and I_NMDA_ components.

### Wnt-5a-induced potentiation increases both the Pr and the quantal amplitude of I_NMDA_ in silent synapses

**FIGURE 2:**
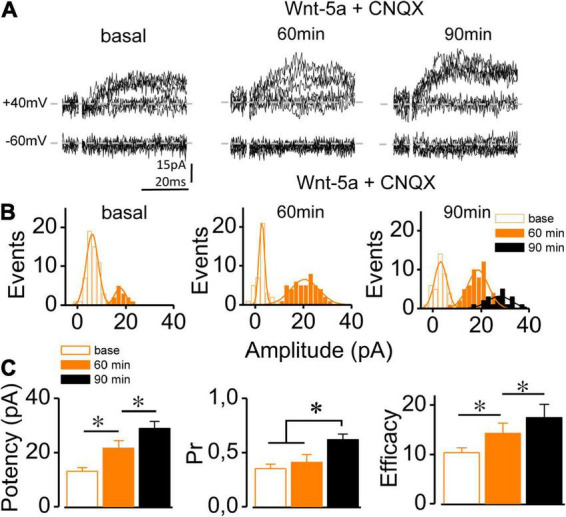
Wnt-5a-induced conversion is independent of AMPAR activation. **(A)** Superimposed meEPSC traces (10 sweeps) recorded at –60 and + 40 mV in a single silent synapse at baseline and 60 and 90 min after Wnt-5a perfusion in the presence of CNQX (20 μM). **(B)** Representative cumulative histogram of meEPSC amplitude at + 60 mV before (basal) and after 60 and 90 min of Wnt-5a plus CNQX treatment showing failures (white bars) and successes (orange-black bars). **(C)** The average change in the synaptic potency, Pr, and synaptic efficacy at baseline and after 60 and 90 min of Wnt-5a plus CNQX, measured at + 40 mV. Statistics: Error bars indicate ± SEM, *t*-test (*p < 0.05).

A previous report on functional synapses showed that the ligand Wnt-5a increased in both I_AMPA_ and I_NMDA_ (Cerpa et al., 2010, 2011, 2015). However, it is unknown whether the potentiation of each component occurs independently of the other in immature synapses. To assess whether the Wnt-5a-induced insertion of AMPAR is required to induce the potentiation of I_NMDA_, silent synapses were perfused with CNQX (20 μM) for 15–20 min before the addition of Wnt-5a. During the baseline period and 15 min after the addition of CNQX, no meEPSCs were displayed at –60 mV, showing the complete absence of I_AMPA_ throughout the experiment (Supplementary Figure 3). In the presence of CNQX, I_NMDA_ increased 60 min after Wnt-5a addition (Figure 2A). This increase can be separated into two temporal stages following the distribution of the events (Figure 2B). The first stage, 60 min in the presence of Wnt-5a (14.07 ± 8.1 pA), and the second stage, 90 min after the addition of Wnt-5a, showed two superimposed curves of amplitude distribution (18.87 ± 2.1 pA and 27.57 ± 6.1 pA; ∼100 trials: Figure 1B). In addition, the mean synaptic potency of NMDAR-mediated meEPSCs progressively increased with the addition of Wnt-5a (basal: 12.95 ± 1.51 pA; 60 min: 21.55 ± 2.79 pA; 90 min: 28.96 ± 2.50 pA; *n* = 5; *p* = 0.035, Figure 2C). The transmission failures at 90 min were lower in comparison to both the basal condition and the condition after 60 min, as observed by the curves that peaked at approximately 0 pA (Figure 2B). Although the basal Pr did not change after 60 min of treatment (basal: 0.35 ± 0.04 vs. 60 min: 0.41 ± 0.07, *n* = 5; *p* = 0.075), it increased at a later stage (90 min: 0.62 ± 0.05, *n* = 5; *p* = 0.0035; Figure 2C). The mean synaptic efficacy showed an increase after 60 min (10.33 ± 1.03 pA vs. 14.35 ± 2.08 pA, respectively) and remained increased up to 90 min (17.51 ± 2.71 pA; *n* = 5; *p* = 0.023, Figure 2C). The above results are consistent with the idea that strengthening of the I_NMDA_ component induced by the ligand Wnt-5a does not require the activation of AMPARs. These progressive changes in the potency and Pr of NMDAR-mediated responses occurred in parallel, suggesting a coordinated mechanism between the postsynaptic and presynaptic components.

### Wnt-5a triggers conversion through both N-methyl-D-aspartate receptor-dependent and N-methyl-D-aspartate receptors-independent mechanisms

**FIGURE 3:**
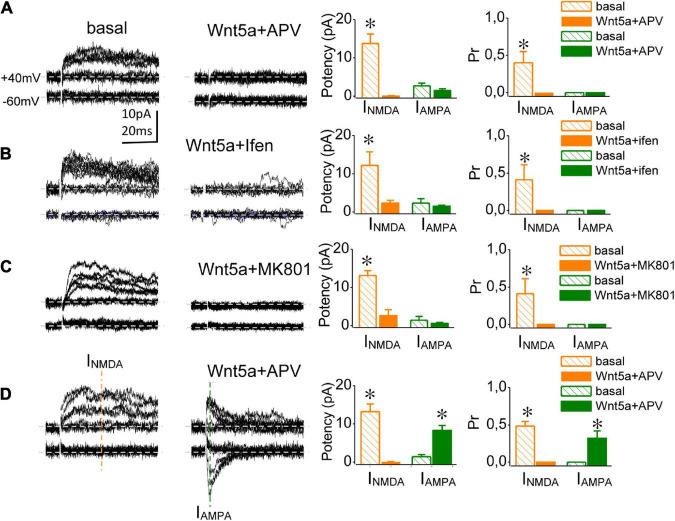
Wnt-5a-induced conversion occurs by both NMDAR-dependent and NMDAR-independent mechanisms. **(A)** meEPSC traces from representative NMDA-independent silent synapses (converted group) recorded at baseline and **(right)** 60 min after Wnt-5a perfusion in the presence of APV (20 μM, **left**). Average change in the synaptic potency and Pr from I_NMDA_ (orange) and I_AMPA_ (green) meEPSC components obtained at baseline and after 60 min of Wnt-5a plus APV (**lower graphics**, respectively). **(B)** meEPSC traces from representative NMDA-dependent silent synapses (unconverted group) recorded at baseline **(right)** and 60 min after Wnt-5a perfusion plus APV **(left)**. Average change in the synaptic potency and Pr of I_NMDA_ (orange) and I_AMPA_ obtained at baseline and after 60 min of Wnt-5a plus APV (**lower graphics**, respectively). **(C)** Superimposed meEPSC traces recorded at baseline **(right)** and 60 min after Wnt-5a perfusion in the presence of ifenprodil (10 μM; **left**). The average change in the synaptic potency and Pr at baseline and after 60 min of Wnt-5a plus ifenprodil, recorded at –60 and + 40 mV (**lower graphics**, respectively). **(D)** Superimposed meEPSC traces were recorded at baseline **(right)** and 60 min after perfusion with MK-801 (40 μM) plus Wnt-5a **(left)**. Average change in the synaptic potency and Pr at baseline and after 60 min of Wnt-5a plus MK-801, recorded at –60 and + 40 mV (**lower graphics**, respectively). Statistics: Error bars indicate ± SEM, *t*-test (*p < 0.05).

Classically, the conversion of silent synapses requires NMDAR activation (Isaac et al., 1995). However, several forms of NMDAR-independent AMPAR insertion have been described thus far (Fortin et al., 2012; Volianskis et al., 2015). Therefore, we assessed whether Wnt-5a-induced conversion is independent of NMDAR activation. The addition of APV (25 μM) completely abolished I_NMDA_ (Supplementary Figure 4). Sixty minutes of perfusion with Wnt-5a in the presence of APV produced two outcomes: (i) a population of silent synapses did not convert in presence of NMDA inhibitors (or were unconverted; Figures 3A–C, respectively) and ii) a population of silent synapses became functional in a manner independent of NMDA receptor activation (or converted; Figure 3D). Under basal conditions, both the unconverted (69%) and converted (31%) groups displayed only I_NMDA_-mediated meEPSCs at + 40 mV, showing similar mean values of synaptic potency (unconverted: 13.75 ± 2.5 pA; *n* = 4; vs. converted: 13.23 ± 1.8 pA; *n* = 5; *p* = 0.083 Figures 3A–D, left graphic) and the Pr (unconverted: 0.41 ± 0.06, *n* = 4 vs. converted: 0.48 ± 0.1; *n* = 5; *p* = 0.069; Figures 3A,B, right graphics). After 60 min in the presence of APV plus Wnt-5a, in the unconverted group the synaptic potency of I_NMDA_-mediated responses fell from 13.75 ± 2.5 pA at baseline to 0.01 ± 0.01 pA (60 min, *n* = 5), whereas the Pr decreased from 0.41 ± 0.05 to zero, corroborating the blockade of I_NMDA_ (Figure 3A, right panel). Instead, after 60 min the converted group displayed meEPSCs mediated by I_AMPA_ at both –60 and + 40 mV of Hp. The successes evoked at depolarized potentials showed only the fast, I_AMPA*R*_-mediated component, with no slow I_NMDA_ APV-sensitive component (Figure 3D). In the presence of Wnt-5a, the synaptic potency of I_AMPA_ at –60 mV increased from 0 to 8.35 ± 1.5 pA (*n* = 5), while at an Hp of + 40 mV (5.89 ± 1.6 pA; *n* = 4; *p* = 0.061; Figure 3D, right panel). The Pr of I_AMPA_-mediated meEPSCs showed similar values at both Hp (–60 mV: 0.32 ± 0.1; *n* = 5 vs. + 40 mV: 0.25 ± 0.1; *n* = 4; *p* = 0.05; Figures 3A–D, right panel). Taken together, these findings indicate that Wnt-5a triggers the postsynaptic insertion of AMPARs through both NMDAR-dependent (i.e., the unconverted group and NMDAR-independent mechanisms (i.e., the converted group), which could be associated with different maturation stages of silent synapses.

The immature hippocampus mostly expresses NMDARs rich in GluN2B subunits (Sheng, 2001; Barria and Malinow, 2002; Yasuda and Mukai, 2015). Their activation seems to be a requirement for silent synapse conversion, as has been shown by circuit reactivation in hippocampal cultures (Westbrook et al., 1997; Nakayama et al., 2005). Therefore, we assessed whether Wnt-5a-induced conversion requires the selective activation of GluN2B-subunit-containing NMDARs. Under basal conditions, and as previously shown, silent synapses exhibited only failures at Hp of –60 mV and NMDAR-mediated meEPSCs at a Hp of + 40 mV, with a synaptic potency of 12.2 ± 3.6 pA, *n* = 4, and a Pr of 0.49 ± 0.15, *n* = 4 (Figure 3B, lower graphics). Wnt-5a was added to the perfusion medium after blocking the NMDAR-mediated meEPSCs with ifenprodil (10 μM; Supplementary Figure 5). After 60 min in the presence of Wnt-5a plus ifenprodil, no meEPSCs were detected at either + 40 mV or –60 mV, and the synaptic potency and Pr were close to zero (Figure 3B). Thus, the activation of GluN2B-subunit-containing NMDARs is required for Wnt-5a-induced conversion. Next, we asked whether the AMPAR insertion induced by Wnt-5a in silent synapses requires calcium influx through NMDARs. To test this idea, we added MK-801 and then Wnt-5a. Under basal conditions, silent synapses exclusively exhibited failures at –60 mV and showed successful responses only at + 40 mV, with a synaptic potency of 13.1 ± 1.3 pA and a Pr of 0.296 ± 0.15 (*n* = 3; Figure 3D). Once I_NMDA_ blockade was reached (MK-801 40 μM; Supplementary Figure 5), we tested the effects of the Wnt ligand 60 min later. Thereafter, no successful responses were evoked at either –60 or + 40 mV, reflecting the lack of synaptic conversion (Figure 3D). Both ifenprodil and MK801 inhibited conversion at all synapses at which these inhibitors were tested. This finding could be due to converted synapses NMDA- independent are being scarcer than silent synapses NMDA-dependent. Taken together, these findings suggest that Wnt-5a transforms silent synapses into functional ones used at least two mechanisms, the most prevalent being postsynaptic calcium increase probably mediated by GluN2B-containing NMDARs.

Thus, calcium increase through those receptors likely represents one of the critical steps in the molecular pathways that trigger the insertion of AMPAR.

## Discussion

### Wnt-5a induces synaptic conversion and the emergence of new silent synapses

Unsilencing of glutamatergic synapse is a basic mechanism in developing synaptic plasticity, mainly through presynaptic and/or postsynaptic mechanisms. At the postsynaptic level, the main mechanism of conversion requires the insertion of synaptic AMPAR channels and/or an increase in conductance (i.e., quantal size) (Sanz-Clemente et al., 2013), whereas, at the presynaptic level, the conversion includes an increase in the Pr and/or the number of release sites (i.e., quantal content) (Malinow and Tsien, 1990; Kerchner and Nicoll, 2008). In functional synapses, Wnt-Frizzled signaling promotes the strengthening of synaptic transmission by inducing the recruitment and trafficking of both AMPARs and NMDARs in dendritic spine (Dinamarca et al., 2008; Farias et al., 2009; Cerpa et al., 2010, 2015; McLeod et al., 2018). We showed that Wnt-5a increases synaptic potency in non-functional, immature hippocampal synapses through both AMPAR- and NMDAR-mediated meEPSCs. According to previous pharmacological findings (Varela-Nallar et al., 2010; Cerpa et al., 2011), the AMPAR-mediated conversion of silent synapses induced by Wnt ligands can also be triggered by Foxy-5 and inhibited by the Wnt scavenger sFRP-2. Based on previous evidence from mature synapses, the plastic changes induced by Wnt ligands could be induced by both RoR2 and/or Fz activation (Cerpa et al., 2015; McQuate et al., 2017). Here we observed that until 60 min in the presence of Wnt-5a, the amplitude of I_AMPA_ and I_NMDA_ increased, while the Pr increased only in the I_AMPA_ component. This fact is consistent with incorporating AMPARs more than an increase in the Pr. Interestingly, the longer the exposure to Wnt-5a (i.e., 90 min), the increase in the amplitude of the I_NMDA_ was accompanied by an increase in the Pr (Bolshakov and Siegelbaum, 1995; Hsia et al., 1998). This finding may be explained by the birth of a new silent synapse activated by the same terminal. Accordingly, we showed that Wnt-5a regulates spinogenesis as well as increases of Bassoon clustering, suggesting the concerted participation of Fz receptor in the pre and postsynaptic differentiation during the early development of the hippocampus (Farias et al., 2009; Varela-Nallar et al., 2009; Varela-Nallar et al., 2010). In the time window in which most of the hippocampal circuitry is established (12-day-old animals), the production of the Wnt ligands, which functionally affects the conversion process, should have a mainly neuronal origin. It has been found that neural progenitors of the hippocampus secrete Wnt3A (Lie et al., 2005), and astrocytes also regulate cellular events associated with aging through secretion of Wnt3 (Okamoto et al., 2011).

The increase in the synaptic potency of I_NMDA_ could be explained by either NMDAR insertion (Cerpa et al., 2011; McQuate et al., 2017) or an increase in NMDAR conductance, as described in mature synapses of organotypic hippocampal slices (Sanz-Clemente et al., 2013). However, the contribution to synaptic strengthening attributed to the quantal size (i.e., unitary conductance changes) and/or to an increase in the quantal content (i.e., the number of release sites and/or Pr) (Malinow and Tsien, 1990; Kerchner and Nicoll, 2008; Cabezas and Buno, 2011; Sanz-Clemente et al., 2013) in the present study cannot be ruled out. Likewise, the lower number of failures to NMDAR-mediated responses than AMPAR-mediated responses has been associated with increase of the fraction of silent synapses among total synapses (Isaac et al., 1995; Liao et al., 1995), so that the highest rate of EPSC measured at + 40 mV suggest an increase of silent synapses in the presence of Wnt ligand. However, further electrophysiological and morphological studies will be necessary to identify the pre- and postsynaptic mechanisms implicated in the unsilencing and formation of new synapses.

### Wnt-5a-activated pathways implicated in triggering silent synapse conversion

**FIGURE 4:**
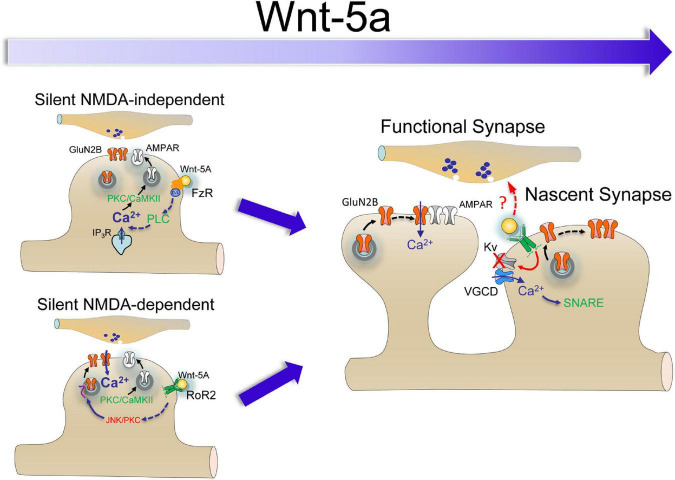
Putative cascades of Wnt-5a-induced conversion of silent synapses. There are two possible activation mechanisms: (1) NMDAR-independent activation or conversion, initiated by FzR activation, promoting Ca^2+^ release by PLC activation, and (2) NMDAR-dependent activation or non-conversion, initiated by RoR2 activation, which increases I_NMDA_ by PKC and JNK activation and, in turn, increases the intracellular Ca^2+^ concentration. At longer exposure times, RoR2 activation induces depolarization, activating voltage-gated Ca^2+^ channels and, in turn, triggering SNARE-dependent trafficking of NMDARs and promoting the emergence of a nascent synapse.

Although the incorporation of AMPARs in silent synapses through long-term potentiation (LTP) induction requires NMDAR activation (Isaac et al., 1995; Liao et al., 1995), several NMDAR-independent mechanisms have been described to insert receptors into glutamatergic synapses (Westbrook et al., 1997; Nakayama et al., 2005; Cabezas and Buno, 2011; Wang et al., 2013). The slow kinetics of Wnt5a-dependent AMPAR and NMDA receptor insertion suggests a distinct mechanism from activity-dependent unsilencing, which occurs by a faster mechanism. We previously showed that LTP-induced by pairing postsynaptic depolarization was larger in slices pretreated with Wnt-5a than ASCF perfused slices (Cerpa et al., 2011). Interestingly, increase in the potency, Pr, and synaptic efficacy of I_NMDAR_ in the presence of CNQX shows that the potentiation is independent of AMPAR activation. However, more than 50% of silent synapses become functional in the presence of APV, which could be explained by Ca^2+^-dependent mechanism alternatives activated at different stages of maturation. That is, AMPAR recruitment of silent synapses requires postsynaptic Ca^2+^ influx for subsequent calcium/calmodulin-dependent protein kinase II (CaMKII) activation (Liao et al., 2001), which Wnt ligands can activate through several mechanisms, including NMDAR-dependent and NMDAR-independent mechanisms (Figure 4; Cerpa et al., 2011, 2015; McQuate et al., 2017). Similar to mature synapses, in the NMDAR-independent group, the binding of Wnt-5a to the Fz receptor can activate PLC, increasing IP3-dependent Ca^2+^ release from the endoplasmic reticulum and activating CaMKII and PKC (Inestrosa and Varela-Nallar, 2015), which seems to be enough to start AMPAR insertion. In the NMDAR-dependent group, RoR2 activation also activates PKC and JNK, being able to increases the channel opening probability and number of NMDARs activated by spontaneous glutamate release (Lan et al., 2001; Metzbower et al., 2019), and then enhances the Ca^2+^ required to activate the Ca^2+^/CaMKII cascade responsible of AMPAR insertion (Poncer et al., 2002; Cerpa et al., 2015; McQuate et al., 2017). But it is important to note that the formation of the Wnt/Fz pair will not only depend on the availability of the ligand, in our experiments applied exogenously but also on the presence of its Fz receptor pair. This generates an additional layer of complexity, due to the diversity of receptors and the stage of development in which they are expressed. An exciting example of this dynamic process is what occurs in the neural tube, where the expression of Fz receptors is a concentration gradient (Fischer et al., 2007). It is possible to determine that the spatial-temporal expression of these receptors suggests an overlap of functions (Fischer et al., 2007). Then, regulation of synaptic conversion function should be considered in the context of the Wnt signaling pathway rather than considering a role of any specific Wnt/Fz pair. In the NMDAR-dependent group, RoR2 activation also activates PKC and JNK, being able to increases the channel opening probability and number of NMDARs activated by spontaneous glutamate release (Lan et al., 2001; Metzbower et al., 2019), and then enhances the Ca^2+^ mobilization from the extracellular medium required to activate the Ca^2+^/CaMKII cascade responsible for AMPAR insertion (Poncer et al., 2002; Cerpa et al., 2015; McQuate et al., 2017).

Interestingly, the blockade of Ca^2+^ influx through the NMDAR channel by MK-801 completely inhibited AMPAR insertion, suggesting that this Ca^2+^ source could be sufficient to induce conversion in enriched GluN2B synapses. However, competitive inhibition of the NMDAR ligand-binding site by APV did not affect the converted synapse group. These findings could be explained in part by the predominance of GluN2B subunits in a group of less mature synapses (Gray et al., 2011), in which the AMPAR insertion is still activity-independent and APV-insensitive but ifenprodil- and MK-801-sensitive currents (Barria and Malinow, 2002; Storey et al., 2011). Thus, in the converted group, depolarization induced by RoR2 activation could explain Ca^2+^ mobilization from internal stores in the presence of APV. At the same line, previously we showed that the field potential potentiation induced by Wnt-5a was inhibited in absences of calcium or with blockers of calcium channels (Varela-Nallar et al., 2010), suggesting that unsilencing also could be a calcium-dependent process.

Taken together, our results indicate that the activation of GluN2B-containing NMDARs could depend on synaptic developmental state (Wang et al., 2018). Additionally, NMDA receptors that incorporate the GluN2D subunit have been studied in the context of tonic and evoked transmission processes (von Engelhardt et al., 2015; Hanson et al., 2019). Participation of GluN2D different according to the stage of development, showing, for example, different levels of sensitivity to specific inhibition, which is accompanied by differential inhibition to APV according to the stage of development (Hanson et al., 2019).

Based on the pieces of evidence discussed above, our data support a model (Figure 4) in which Wnt-5a can induce the conversion of silent synapses through at least two activation mechanisms: (1) NMDAR-independent activation, initiated by FzR activation, which promotes Ca^2+^ release from intracellular stores by PLC activation or, alternatively, by a mechanism coupled to the activation of voltage-dependent channels, and (2) NMDAR-dependent activation, mediated by non-canonical RoR2 activation, which increases I_NMDA_ by PKC and JNK activation and, in turn, increases the intracellular Ca^2+^ concentration (Cerpa et al., 2015). At longer exposure times, RoR2 activation could induce depolarization, activating voltage-gated Ca^2+^ channels and, in turn, activating the SNARE-dependent trafficking of NMDARs (McQuate et al., 2017), promoting the emergence of a nascent synapse. If these mechanisms are excluded and if they correspond to synapses in different stages of development, these are questions that should be addressed in future studies.

In conclusion, this study shows that the activation of the Wnt pathway induces the conversion of immature synapses into functional synapses and promotes the birth of synapses, demonstrating the pivotal role of the Wnt pathway in neuronal development. These findings suggest that Wnt-5a and its receptors, which are widely expressed during early development (Farias et al., 2009), could represent the critical signal that triggers entry into operation and the formation of glutamatergic circuits in the whole brain. Moreover, this study identifies Wnt signaling as a therapeutic target for the recovery and restoration of synaptic contacts in mental illnesses that affect glutamatergic communication, such as Alzheimer’s disease and schizophrenia.

## Data availability statement

The original contributions presented in this study are included in the article/Supplementary material, further inquiries can be directed to the corresponding author.

## Ethics statement

The animal study was reviewed and approved by the Ethics and Biosafety Committee of the Universidad de Valparaíso and the Institutional Animal Experimentation Ethics Board and the Science Council (FONDECYT) of Chile.

## Author contributions

CÁ-F, MW, and KM performed the investigations, validation, data curation, and formal analysis. MW, WC, and MF contributed to writing the original draft. NI and WC contributed to study conceptualization, visualization, review, and funding. CB contributed to study conceptualization, investigation, writing, and funding acquisition for this work. All authors contributed to the article and approved the submitted version.
