# Supplementary figures and images for: Wnt-5a induces the conversion of silent to functional synapses in the hippocampus
Source: Front Mol Neurosci. 2022 Oct 28;15:1024034. doi: 10.3389/fnmol.2022.1024034 (PMC9650636; doi:10.3389/fnmol.2022.1024034)

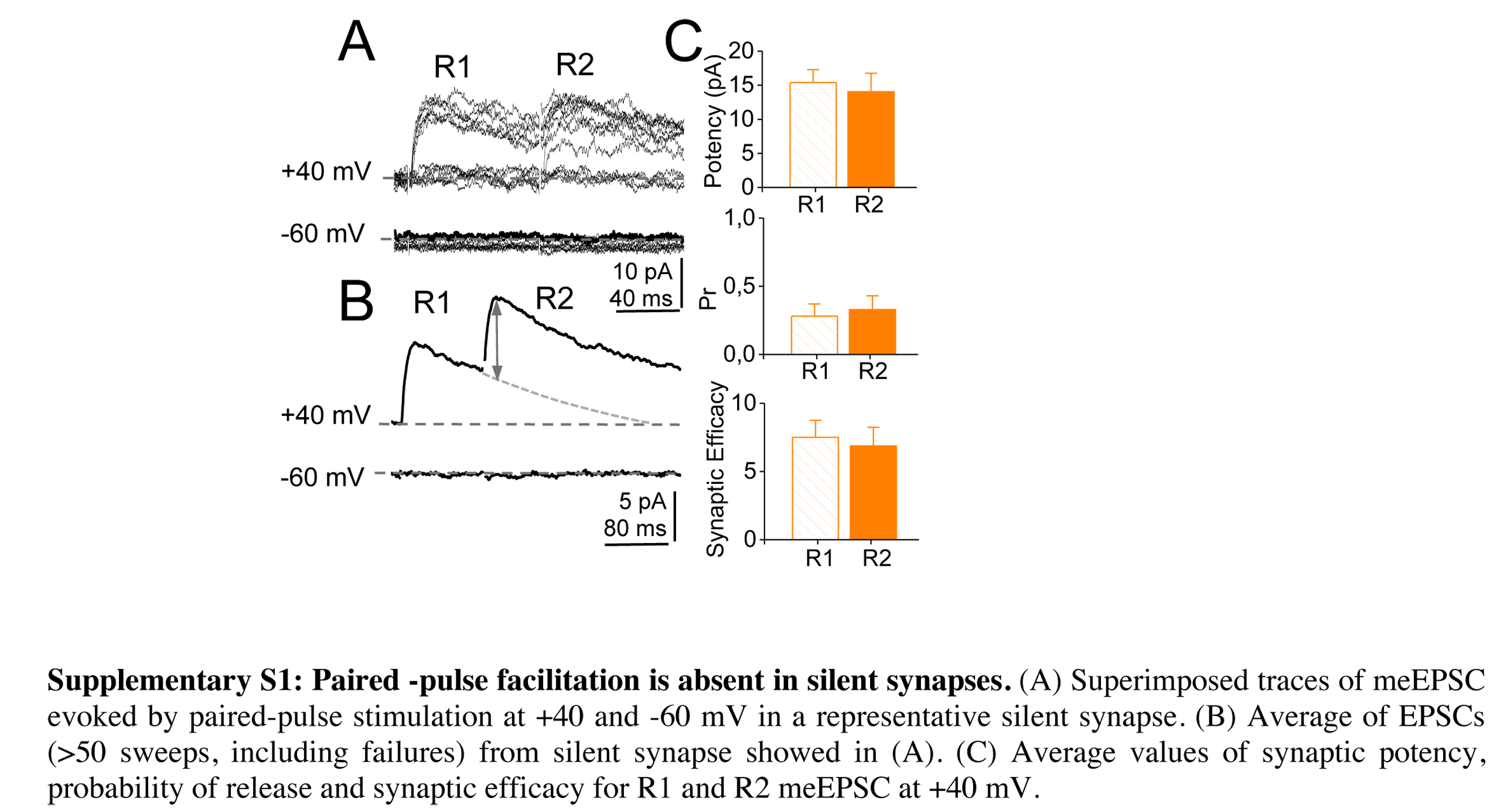

Supplement: Supplementary file 1 [file Image_1.TIF]

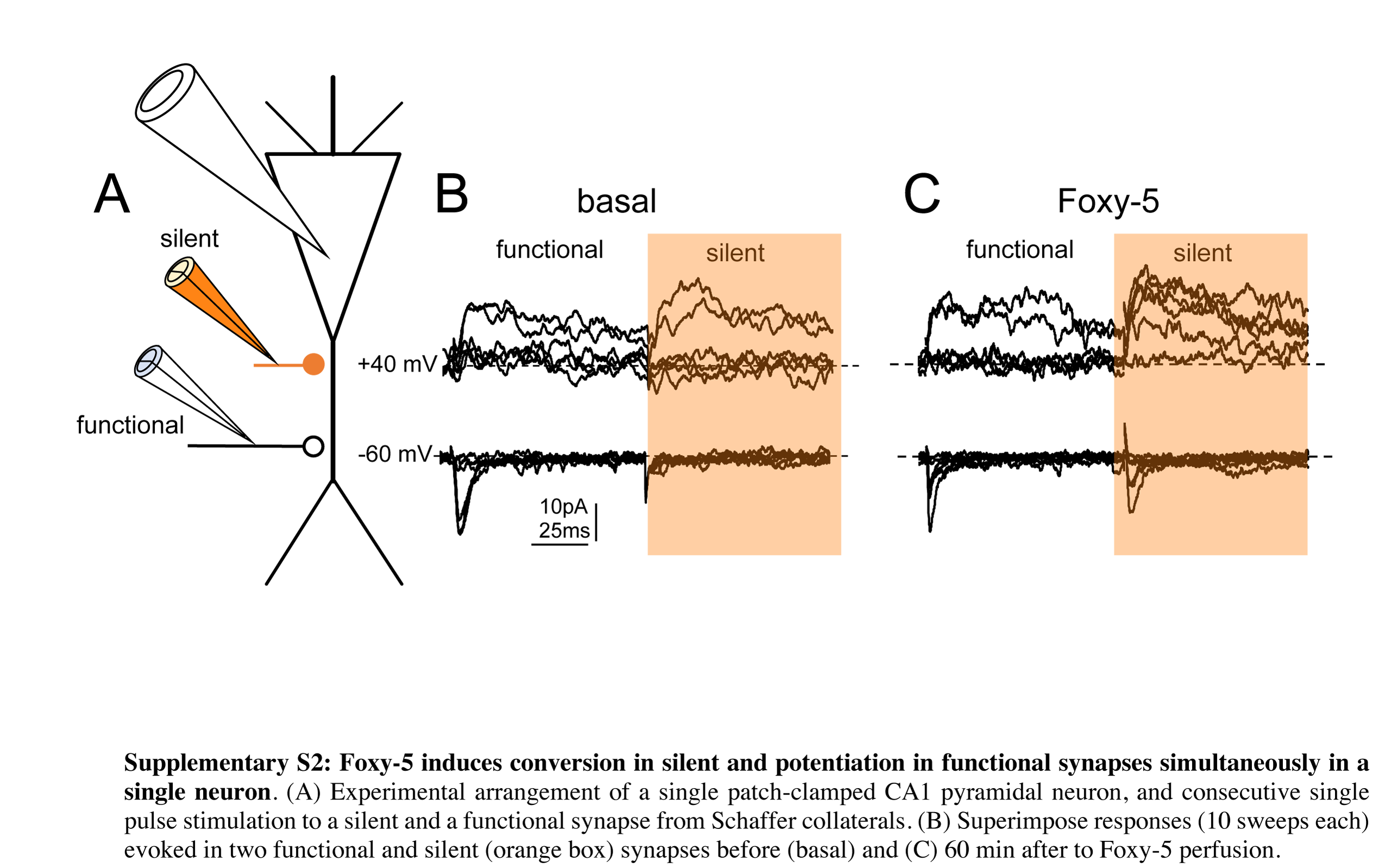

Supplement: Supplementary file 2 [file Image_2.TIF]

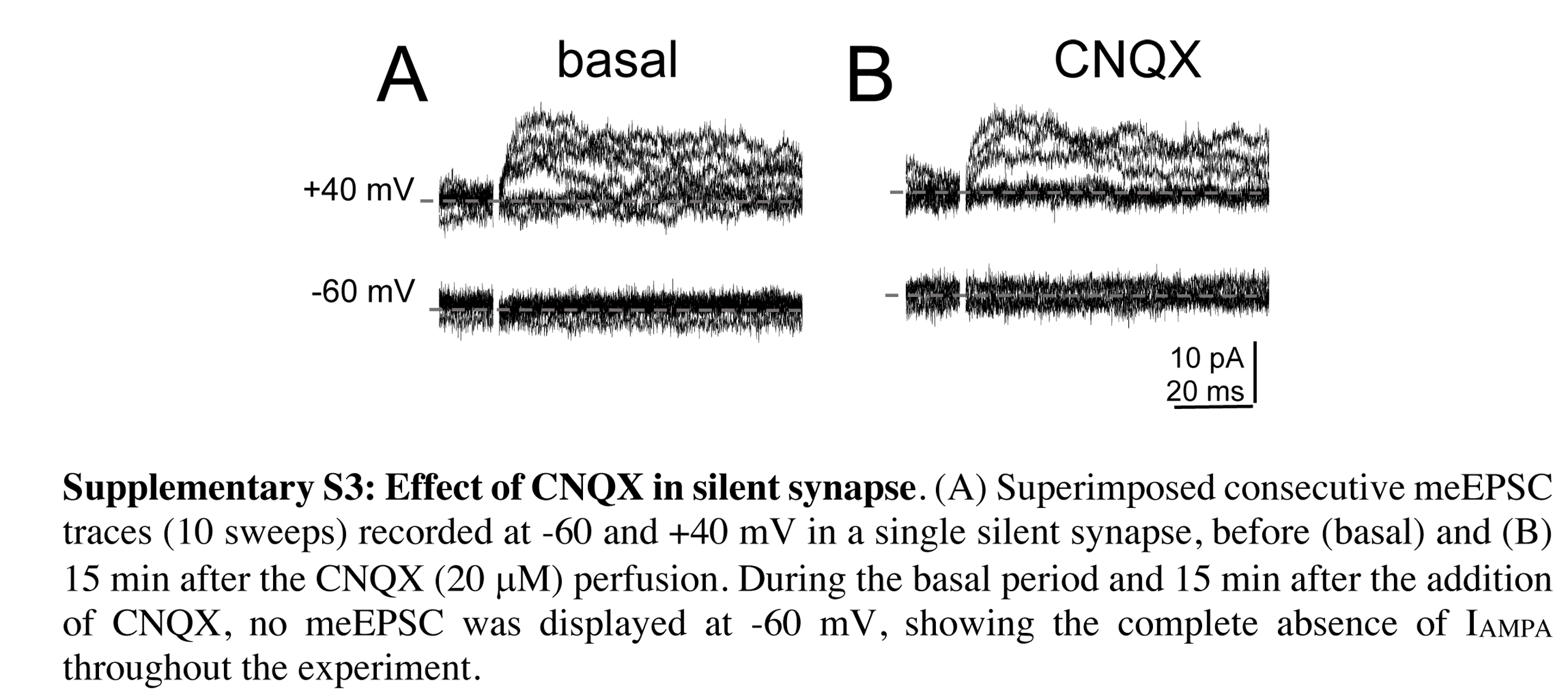

Supplement: Supplementary file 3 [file Image_3.TIF]

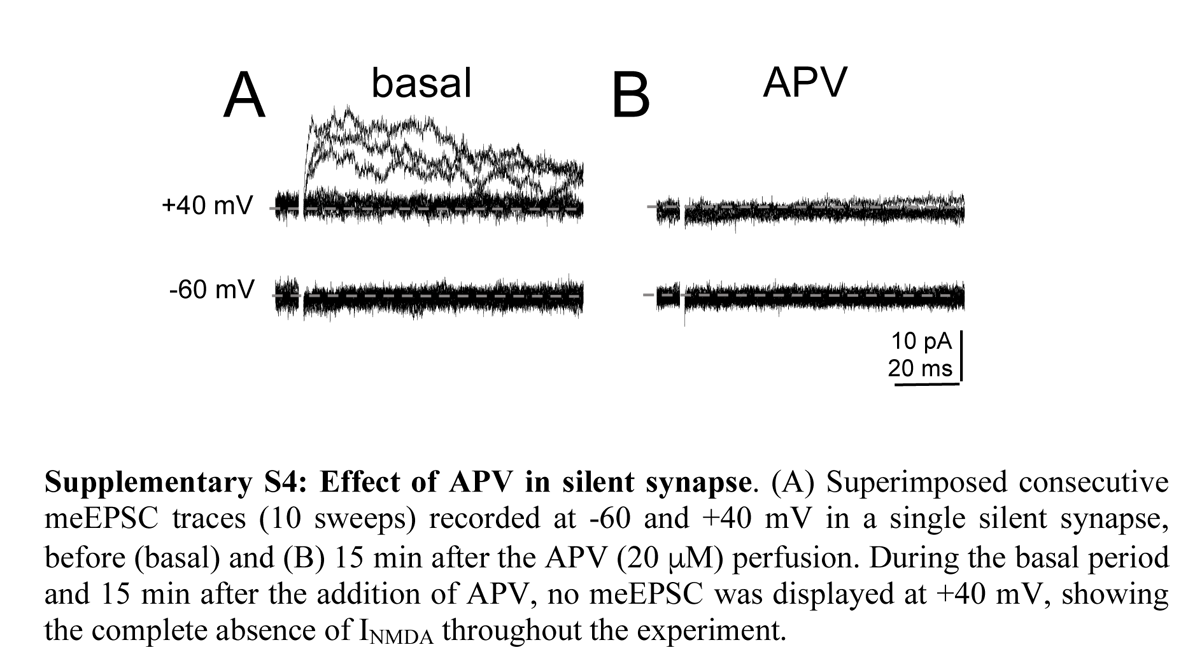

Supplement: Supplementary file 4 [file Image_4.TIF]

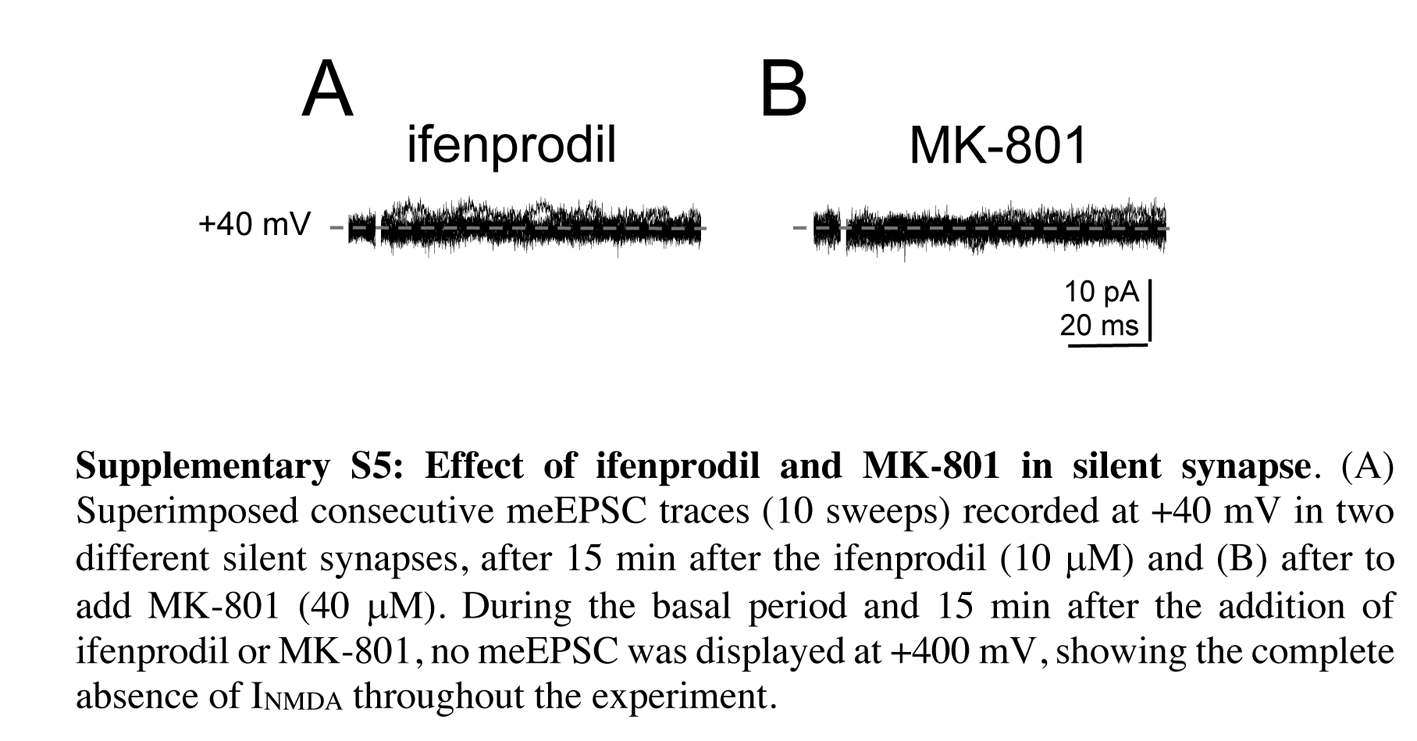

Supplement: Supplementary file 5 [file Image_5.tif]

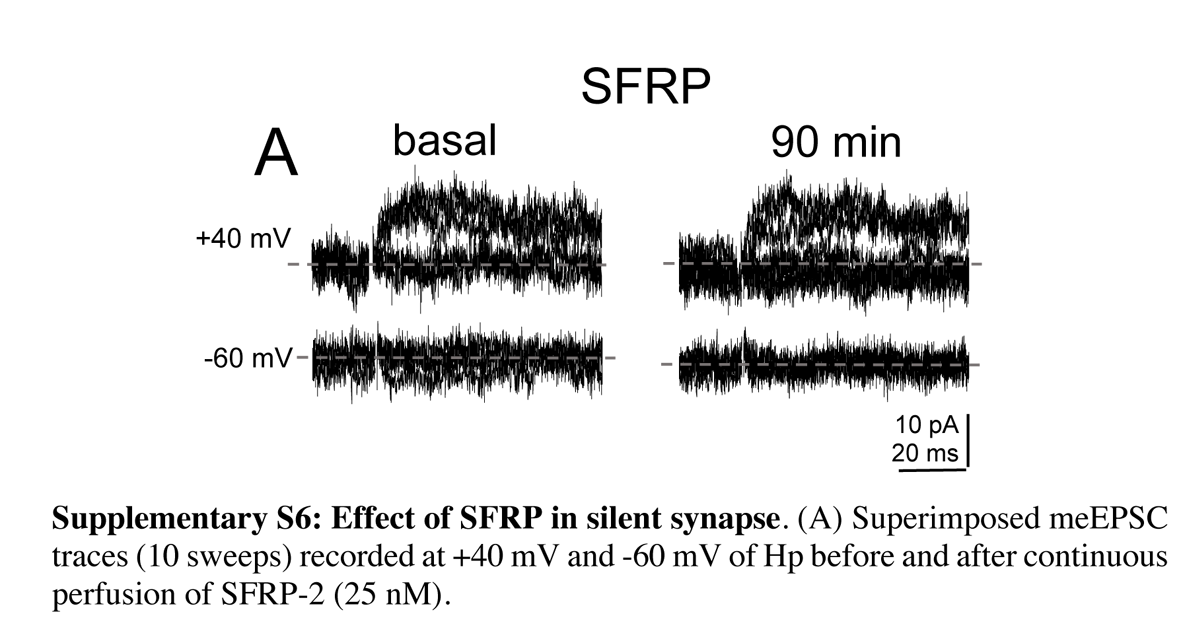

Supplement: Supplementary file 6 [file Image_6.TIF]
